# Follicle-Stimulating Hormone Regulates *igfbp* Gene Expression Directly or *via* Downstream Effectors to Modulate Igf3 Effects on Zebrafish Spermatogenesis

**DOI:** 10.3389/fendo.2017.00328

**Published:** 2017-11-20

**Authors:** Diego Safian, Henk J. G. van der Kant, Diego Crespo, Jan Bogerd, Rüdiger W. Schulz

**Affiliations:** ^1^Reproductive Biology Group, Division Developmental Biology, Institute of Biodynamics and Biocomplexity, Department of Biology, Faculty of Science, University of Utrecht, Utrecht, Netherlands; ^2^Institute of Marine Research, Bergen, Norway

**Keywords:** follicle-stimulating hormone, Igf3, Igf-binding proteins, spermatogonia, differentiation

## Abstract

Previous work showed that pharmacological inactivation of Igf-binding proteins (Igfbps), modulators of Igf activity, resulted in an excessive differentiation of type A undifferentiated (A_und_) spermatogonia in zebrafish testis in tissue culture when Fsh was present in the incubation medium. Using this testis tissue culture system, we studied here the regulation of *igfbp* transcript levels by Fsh and two of its downstream effectors, Igf3 and 11-ketotestosterone (11-KT). We also explored how Fsh-modulated *igfbp* expression affected spermatogonial proliferation by adding or removing the Igfbp inhibitor NBI-31772 at different times. Fsh (100 ng/mL) decreased the transcript levels of *igfbp1a, -3*, and *-6a* after 1 or 3 days, while increasing *igfbp2a* and *-5b* expression, but only after 5 days of incubation. Igf3 down-regulated the same *igfbp* transcripts as Fsh but with a delay of at least 4 days. 11-KT increased the transcripts (*igfbp2a* and *5b*) that were elevated by Fsh and decreased those of *igfbp6a*, as did Fsh, while 11-KT did not change *igfbp1a* or *-3* transcript levels. To evaluate Igfbps effects on spermatogenesis, we quantified under different conditions the mitotic indices and relative section areas occupied by the different spermatogonial generations (type A_und_, type A differentiating (A_diff_), or type B (B) spermatogonia). Igf3 (100 ng/mL) increased the area occupied by A_diff_ and B while decreasing the one for A_und_. Interestingly, a concentration of Igf3 that was inactive by itself (25 ng/mL) became active in the presence of the Igfbp inhibitor NBI-31772 and mimicked the effect of 100 ng/mL Igf3 on spermatogonia. Studies exploiting the different dynamics of *igfbp* expression in response to Fsh and adding or removing NBI-31772 at different times showed that the quick downregulation of three *igfbp* as well as the delayed upregulated of two *igfbps* all support Igf3 bioactivity, namely the stimulation of spermatogonial differentiation. We conclude that Fsh modulates, directly or *via* androgens and Igf3, *igfbp* gene expression, supporting Igf3 bioactivity either by decreasing *igfbp1a, -3, -6a* or by increasing *igfbp2a* and -*5b* gene expression.

## Introduction

In vertebrates, the brain–pituitary system is the major regulator of spermatogenesis and coordinates the activities of somatic cell types in the testis. These activities include the modulation of spermatogonial stem cell (SSC) fate (1–3). The SSCs can self-renew or differentiate, depending on the signaling environment produced by Sertoli cells (SCs) and other somatic cell types (4, 5). Follicle-stimulating hormone (Fsh) regulates the activity of SCs, which then communicate with germ cells *via* short-range signaling. In fish, the *fshr* is expressed not only by SCs but also by Leydig cells (LCs), thus stimulating for example androgen and insulin-like peptide 3 (Insl3) production (6, 7). In eel and zebrafish, recombinant Fsh-induced spermatogonial proliferation and differentiation by stimulating androgen production (8, 9). In zebrafish, Fsh also promoted spermatogenesis in an androgen-independent manner by promoting Igf3 (9) and Insl3 (7), by suppressing anti-Müllerian hormone signaling (10), and by modulating the Notch, Wnt, and Hedgehog signaling systems (11).

IGF signaling promotes proliferation and differentiation of many different cell types across animal species. In most vertebrates, the IGF signaling system is composed of two ligands (IGF1 and 2), two IGF1 receptors (IGF1R1 and 2), and six IGF-binding proteins (IGFBP1-6) (12). Systemic IGFs are mainly secreted by the liver, controlled by growth hormone (GH), but IGFs are also produced locally in many tissues (13).

IGF signaling modulates spermatogenesis in a wide range of animals. Insulin/IGF signaling regulates early stages of male germ cell development (14–16). In mice, the combined knockout of insulin and IGF1 receptors strongly reduced testis size as a consequence of decreased SC proliferation and the resulting reduction of the germ cell supporting capacity (17). A more recent report indicated that blocking the IGF1 receptor in primary cultures of mouse SSCs reduced their proliferation and decreased their colonization capacity when injected in busulfan-treated recipients (18). In rainbow trout, *igf1* and *igf1r* expression was found in cell fractions enriched in SCs but also in spermatogonia and primary spermatocytes (19), while *igf1* expression was restricted to cysts containing spermatogonia in sea bass (20). Furthermore, primary tissue culture studies using prepubertal eel testis showed that IGF was required as permissive factor for the androgen-stimulated differentiating proliferation of spermatogonia (21).

An additional ligand member of the Igf family, Igf3, has been identified in fish gonads (22). In zebrafish testis, Fsh increased *igf3* but not *igf1, 2a*, or *2b* transcript levels, and recombinant zebrafish Igf3 increased the proliferation activity of A_und_ and A_diff_ spermatogonia and upregulated the expression of *dazl*, a marker for type B spermatogonia and spermatocytes (9). Also, Igfbps appear to be relevant for testis function in zebrafish (23). Igfbps bind Igfs with high affinity, thereby inhibiting or potentiating Igf actions (12, 24). Similar to *igfs*, the *igfbp*s are expressed in several tissues, suggesting that local Igfbps can modulate systemic but in particular locally produced Igfs (12). Fsh and triiodothyronine (T_3_), another regulator of *igf3* expression (25), modulated the expression of selected *igfbp*s, and interestingly, adding an Igfbp inhibitor further shifted spermatogonial development toward differentiation at the expense of A_und_ spermatogonia (23). This observation suggested that Igfbps play important roles in modulating spermatogonial proliferation and differentiation behavior.

Here, we studied the transcriptional regulation of the nine zebrafish *igfbps* that are all expressed in testis tissue, by examining the effects of Fsh and of two downstream mediators of Fsh action in the testis, Igf3, and 11-KT. We also report the effects of Igf3 on spermatogonial proliferation and the area occupied by spermatogonia in zebrafish testis, under basal conditions or in the presence of an Igfbp inhibitor. Finally, we have started exploring a potential, functional differentiation among the Igfbps in the zebrafish testis.

## Materials and Methods

### Animals

Adult male zebrafish between 4 and 12 months of age were used in this study. Six to eight animals were used per experiment. All experiments carried out in this study followed the Dutch National regulations for animal care and use in experimentation, and the experimental protocols have been submitted to, and were approved by, the Utrecht University Experimental Animal Committee (2015.I.857.013 and AVD108002015333).

### Tissue Culture

To study the regulation of *igfbp* transcript levels, adult zebrafish testes were dissected for tissue culture experiments using a previously described system (26), in which one testis was incubated under control conditions, the other testis under experimental conditions.

Zebrafish testes were incubated for 5 days under basal conditions or in the presence of recombinant zebrafish Fsh (25, 50, 100, or 1,000 ng/mL) (6). In a second series of experiments, zebrafish testes were incubated under basal conditions or in the presence of Fsh (100 ng/mL) for 1, 3, 5, or 7 days.

To study the effect of Igf3 on *igfbp* expression, zebrafish testes were incubated in the absence or presence of recombinant zebrafish Igf3 (100 ng/mL) (9) for 3 or 7 days. Based on the slow effect of Igf3 on *igfbp* expression, testes were incubated for 5 or 7 days in the presence of Fsh (100 ng/mL) with or without NVP-AEW541 [10 µM; Selleckchem (25)], an inhibitor of Igf1 receptors; incubation media for the control and experimental groups contained the same final concentration of dimethyl sulfoxide (0.1%).

In a different set of experiments, zebrafish testes were incubated under basal conditions or in the presence of 11-KT [200 nM in ethanol (0.01%); Sigma] for 3 or 7 days (10), or in the presence of 11-KT (200 nM) with or without 10 µM NVP-AEW541 for 7 days. The reason to carry out this experiment was the previously reported, slight stimulatory effect of 11-KT on *igf3* transcript levels (9). At the end of the incubation period, testis tissue was snap-frozen in liquid nitrogen and stored at −80°C until RNA extraction.

We have reported previously that 100 ng/mL Igf3 stimulated the proliferation of type A spermatogonia and increased the transcript levels of marker genes associated with spermatogonial differentiation (9). To further study Igf3 effects and the role of Igfbps on zebrafish spermatogenesis, we incubated testes under basal conditions or in the presence of 25 or 100 ng/mL Igf3 for 3 days. The lower dose was expected to have no/little effect on its own, based on a previous dose-response study (9). In addition, zebrafish testes were incubated for 3 days in the presence of 25 ng/mL Igf3 with or without NBI-31772 (10 µM; Sigma-Aldrich), an Igfbp inhibitor (27, 28); NBI-31772 alone has no effects on spermatogenesis or expression of germ cells markers (23).

The Fsh time-course experiment showed that two *igfbp* mRNAs were upregulated with a delay of at least 3 days, so that two experiments were designed to preferentially study these two *igfbps* upregulated by Fsh. To this end, zebrafish testes were incubated for 7 days with 100 ng Fsh/mL in both control and experimental groups. During the last 4 days, the experimental group was in addition exposed to 10 µM NBI-31772. In the second experiment, testes of the control group were incubated in the presence of 100 ng/mL Fsh and 10 µM NBI-31772 for 7 days, whereas in the experimental group, testes were incubated for the first 3 days under the same conditions but for the remaining 4 days, the medium contained only Fsh but no NBI-31772. At the end of the 7 days long incubation period, testis tissue was fixed for morphological analyses.

The production of biologically active steroids was blocked by including trilostane (25 µg/mL; Chemos), an inhibitor of 3β-hydroxysteroid dehydrogenase activity, in all experiment with Fsh, a potent steroidogenic hormone in fish (6).

### Gene Expression

The relative transcript levels of *igfbp*s, germ cells markers, and other genes of interest (Table 1) were analyzed by real-time, quantitative polymerase chain reaction (qPCR) assays. The expression of *igfbp3* was analyzed using a commercial available TaqMan gene expression assay (Applied Biosystems, Cat# 4351372).

**Table 1:** Primers used for gene expression studies.

Target genes	Primers name	Sequence (5′–3′)	Gene information
*igfbp1a*	4194 (Fw)	GAGCCCCGAGCCTAACCA	Safian et al. (23)
	4196 (Rv)	TCTCATAACGGGCCGACG	
*igfbp1b*	4199 (Fw)	GTGGAGCACCACCCTACTGAAG	Safian et al. (23)
	4200 (Rv)	TGCATCACCTGCTGAGCC	
*igfbp2a*	4206 (Fw)	GACCCTAAAGCACCACATGCTAA	Safian et al. (23)
	4207 (Rv)	TTGACCAGGTGCTGGAAAGG	
*igfbp2b*	4211 (Fw)	GCCCACCATGACCAACCA	Safian et al. (23)
	4213 (Rv)	GAAGTAAATGGCACGCGGTC	
*igfbp5a*	4226 (Fw)	CTCCCCTTCCCATCGACAA	Safian et al. (23)
	4227 (Rv)	CAGAAGGAAGCTGGACGGAAT	
*igfbp5b*	4333 (Fw)	CGCAAACATGTAAGCCCTCTAG	Safian et al. (23)
	4334 (Rv)	ATGGAGTTCAAATGCCGGG	
*igfbp6a*	4955 (Fw)	CCTCTGGTGGCGACAAATATG	Safian et al. (23)
	4956 (Rv)	TGCATCAACTGCCAGAACTCTAA	
*igfbp6b*	4928 (Fw)	TGACATCTACATCCCAAACTGTGA	Safian et al. (23)
	4929 (Rv)	GGAAAAAGCAGTGTCGGTCC	
*foxa2*	5741 (Fw)	GTCAAAATGGAGGGACACGAAC	Potential marker for type A undifferentiated spermatogonia
	5743 (Rv)	CATGTTGCTGACCGAGGTGTAA	
*piwil2*	2994 (Fw)	TGATACCAGCAAGAAGAGCAGATCT	Expressed in all germ cell type except type A_und_ spermatogonia and spermatozoa (29)
	2995 (Rv)	ATTTGGAAGGTCACCCTGGAGTA	
*dazl*	3104 (Fw)	AGTGCAGACTTTGCTAACCCTTATGTA	Expressed mainly in type B spermatogonia and primary spermatocytes (30)
	3105 (Rv)	GTCCACTGCTCCAAGTTGCTCT	
*igf1ra*	2362 (Fw)	TACATCGCTGGCAACAAGCA	Igf1 receptor a (30)
	2363 (Rv)	TCATTGAAACTGGTCCTTATGCAAT	
*igf1rb*	2595 (Fw)	GTGCTGGTCCTCTCCACACTCT	Igf1 receptor b (30)
	2596 (Rv)	TTACCGATGTCGTTGCCAATATC	

*Fw, forward; Rv, reverse*.

Total RNA was isolated from the tissue using an RNAqueous Micro kit (Ambion), according to the manufacturer’s protocol. cDNA synthesis from total RNA and quantification of transcript levels were carried out as described previously (31). In brief, 2 µg of total RNA were reverse transcribed using 250 U of Supercript II RNase-reverse transcriptase (Life Technologies). qPCR were performed by using 2× SYBER Green assay mix (Applied Biosystems), specific qPCR primers (900 nM) and 5 µL of cDNA in a total volume of 20 µL. The quantification cycle (Cq) values were determined in a Step One Plus Real-Time PCR System (Applied Biosystems) using default settings. The relative amounts of mRNA in the cDNA samples were calculated using the arithmetic comparative method (ΔΔCt method), according to Bogerd et al. (31). Expression of the *ribosomal RNA 18S* (*18S*) transcript was stable (Figure S1 in Supplementary Material). *18S* expression served as reference transcript and was analyzed using a commercially available TaqMan gene expression assay (Applied Biosystems). All results were expressed as fold change with respect to the control group.

### Morphological Analysis

To quantify the proliferation activity of A_und_, A_diff_, and B spermatogonia, 100 µg/mL of the proliferation marker 5-bromo-2′-deoxyuridine (BrdU; Sigma-Aldrich) was added to the tissue culture medium during the last 6 h of the incubation period. After fixation in methacarn (60% [v/v] absolute ethanol, 30% chloroform, and 10% acetic acid), the samples were dehydrated in graded ethanol (70, 96, and 100%), embedded in Technovit 7100 (Heraeus Kulzer) and sectioned at a thickness of 4 µm. To determine the proliferation activity, one set of sections was used to localize BrdU as described previously (26). The mitotic index was determined by analyzing 100 spermatogenic cysts (A_diff_ and B spermatogonia) or 100 A_und_ cells, discriminating between BrdU positive and negative cysts/cells, respectively.

To quantify the proportion of section area occupied by the different spermatogonial cell types, another set of sections was stained with toluidine blue and 10 randomly chosen, non-overlapping fields were photographed at ×400 magnification with a digital camera. The images were analyzed quantitatively based on the number of points counted over the germ cell types investigated (A_und_, A_diff_, and B spermatogonia), using the ImageJ freeware (National Institutes of Health, Bethesda, MD, USA, http://rsbweb.nih.gov/ij) with a 540-point grid.

### Statistical Analysis

Statistical analyses were carried out using the GraphPad Prism 5 software package (San Diego, CA, USA). Since our tissue culture system compares the two testes of a given fish incubated under control versus experimental conditions, we applied Student’s *t*-test for paired observation to estimate statistical significance. All data are presented as fold of basal (mean ± SEM). The individual data before normalization are provided as supplemental figures (Figure S2 in Supplementary Material). To achieve homogeneity of variance, data were log transformed when appropriate.

## Results

### Fsh and Downstream Mediators Modulate igfbp Expression

**Figure 1:**
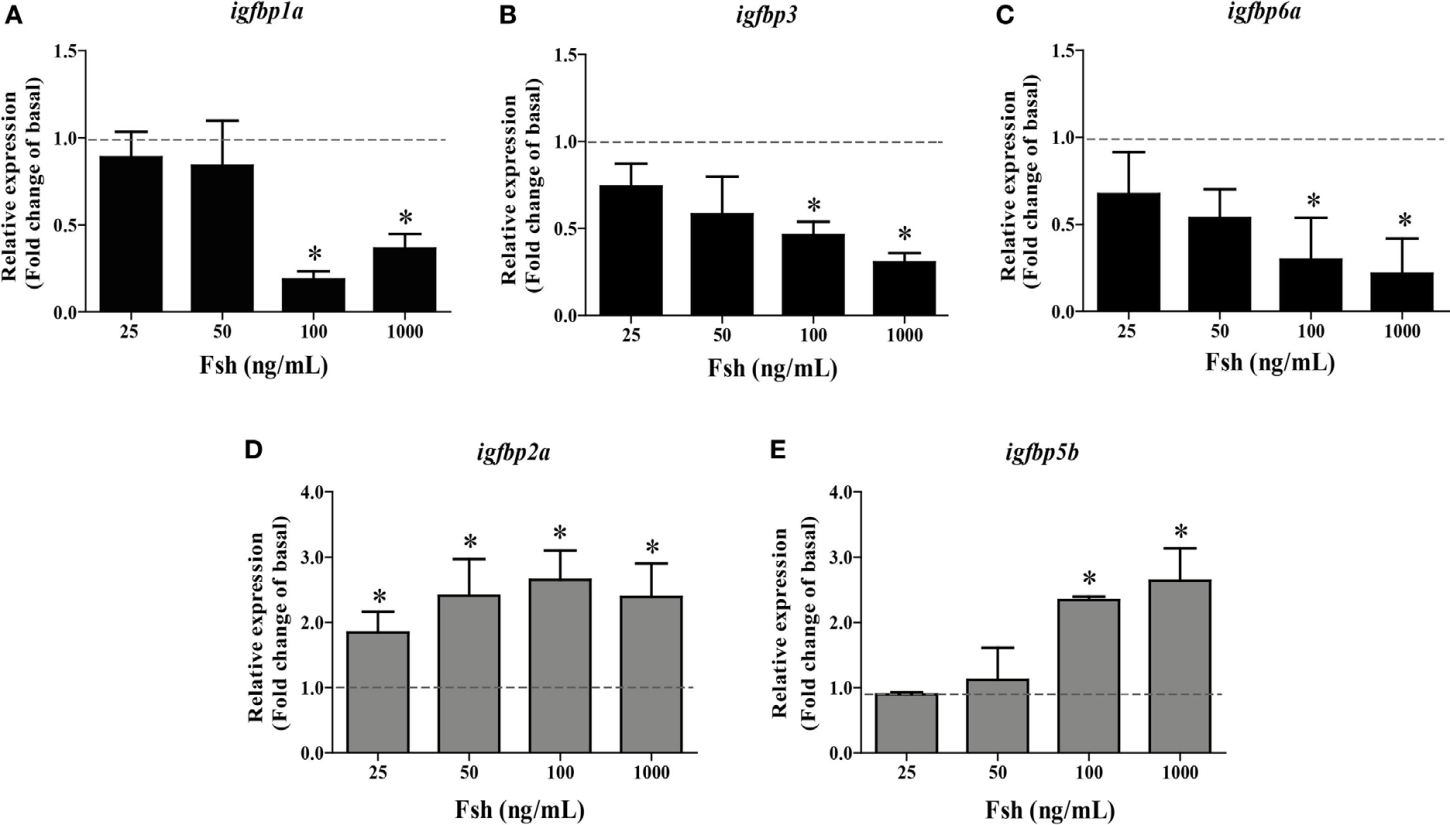
Follicle-stimulating hormone (Fsh) dose-response study on *igfbp* transcript levels in adult zebrafish testis after 5 days of primary organ culture. Fold-change of gene expression of *igfbp1a* **(A)**, *igfbp3* **(B)**, *igfbp6a* **(C)**, *igfbp2a* **(D)**, and *igfbp5b* **(E)** under basal conditions (dotted line) or in the presence of 25, 50, 100, or 1,000 ng recombinant zebrafish Fsh per mL (*n* = 6 for all concentrations) (bars). Asterisks indicate significant differences (*P < 0.05) compared to the respective control group.

To study the regulation of *igfbp* transcript levels by Fsh, dose-response and time-course experiments were carried out. *igfbp1b, 2b, 5a*, and *6b* transcript levels were not regulated by Fsh at any concentration or time evaluated in the present study (data not shown). From the five remaining transcripts, three (*igfbp1a, 3*, and *6a*) were down- and two (*igfbp2a* and *5b*) were upregulated by Fsh. The Fsh dose-response experiment was carried out using 5 days of incubation. The downregulated *igfbp1a, 3*, and *6a* responded to the two higher concentrations of 100 and 1,000 ng/mL Fsh (Figures 1A–C). This was also the case as regards the upregulated *igfbp5b*, while the second upregulated *igfbp2a* responded to all Fsh concentrations used (Figures 1D,E).

**Figure 2:**
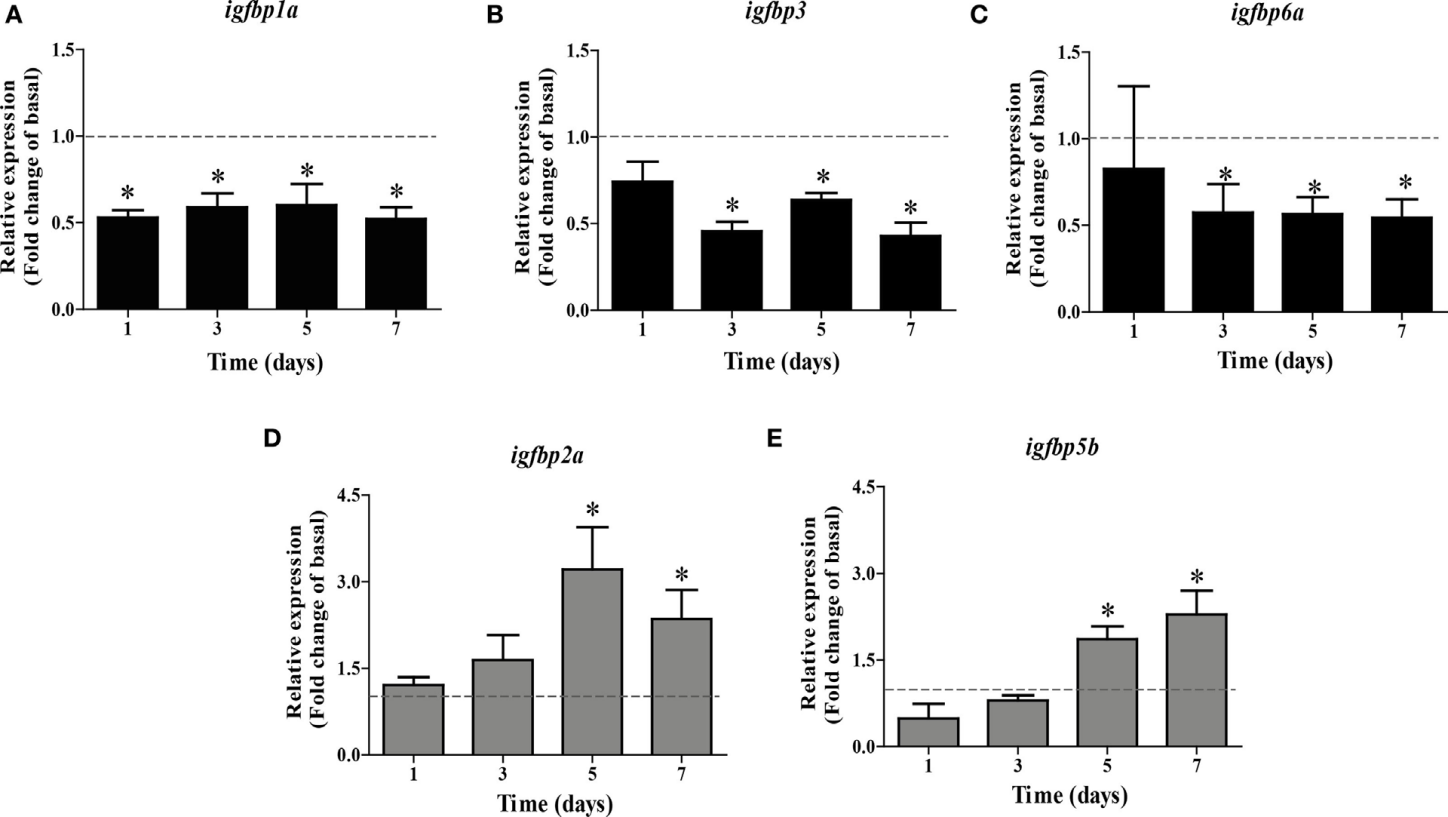
Follicle-stimulating hormone (Fsh) time-course study on *igfbp* transcript levels in adult zebrafish testis after 1–7 days of primary organ culture. Transcript levels of *igfbp1a* **(A)**, *igfbp3* **(B)**, *igfbp6a* **(C)**, *igfbp2a* **(D)**, and *igfbp5b* **(E)** are expressed as fold-change compared to basal conditions (absence of Fsh; represented by a dotted line), as induced by recombinant zebrafish Fsh (100 ng/mL) (bars). The duration of the *ex vivo* culture varied between 1 and 7 days (*n* = 5–7). Asterisks indicate significant differences (*P < 0.05) compared to the respective control group.

Based on these data, we used 100 ng Fsh/mL in the time-course experiment. We found that *igfbp1a* expression was quickly downregulated after 1 day, while *igfbp3* and *6a* transcript levels had decreased significantly after 3 days of incubation (Figures 2A–C). Upregulation of *igfbp2a* and *5b* required more time and became significant after 5 days of tissue culture (Figures 2D,E).

**Figure 3:**
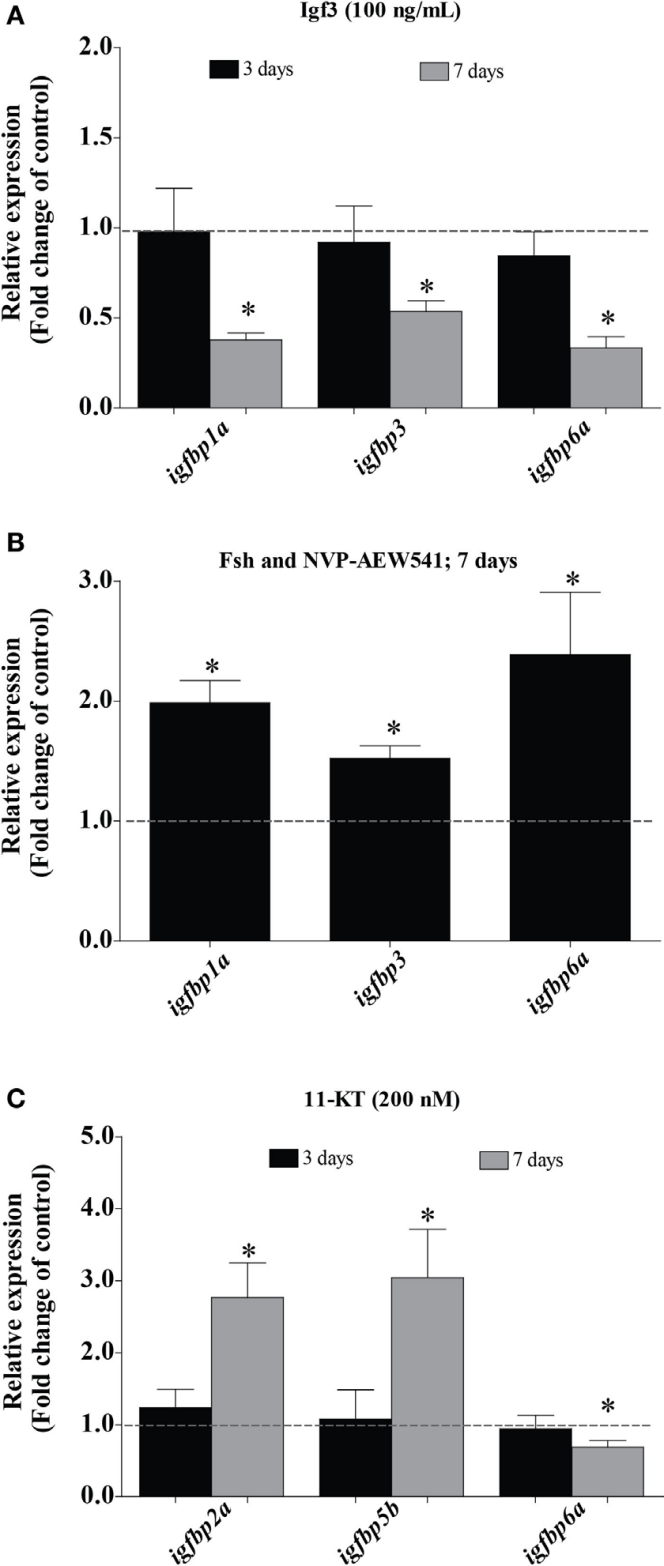
Effect of Igf3 and 11-KT on *igfbp* transcript levels in adult zebrafish testis. **(A)** Transcript levels of *igfbp*s in the presence of Igf3 (100 ng/mL) after 3 or 7 days of incubation (*n* = 8). **(B)** *Igfbp* expression in response to follicle-stimulating hormone (Fsh; 100 ng/mL) in the absence or presence of Igf receptor inhibitor NVP-AEW541 (10 µM) after 7 days of incubation (*n* = 8). **(C)** Transcript levels of *igfbp*s in basal conditions or in the presence of 11-KT (200 nM) after 3 (*n* = 7) or 7 (*n* = 6) days of incubation. Transcript levels of *igfbp*s are expressed as fold-change compared to the respective control condition (basal or 100 ng/mL Fsh) represented by a dotted line. Asterisks indicate significant differences (*P < 0.05) compared to the respective basal group.

Since Fsh increased Igf3 release, we studied if (some of) the changes induced by Fsh are mediated by Igf3. As shown in Figure 2, Fsh modulated the transcript levels of some *igfbps* after a short (1 or 3 days for *igfbp1a, igfbp3*, and *igfbp6a*) and others after a longer (5 or 7 days for *igfbp2a* and *igfbp5b*) period of incubation. Therefore, testes were incubated in the presence of recombinant zebrafish Igf3 (100 ng/mL) for 3 or 7 days. Effects of Igf3 on *igfbp* expression were evident after 7 days of incubation only, when we found significantly decreased transcript levels of *igfbp1a, 3*, and *6a* (Figure 3A); *igfbp2a* and *igfbp5b* expression did not change in response to Igf3 after 3 or 7 days of incubation (data not shown). To directly examine if the slow Igf3 effects on *igfbp* transcript levels are downstream of Fsh, we incubated testis tissue for 5 or 7 days with Fsh in the absence or presence of a pharmacological Igf receptor inhibitor. While *igfbp* transcript levels did not change after 5 days (data not shown), all three transcripts (*igfbp1a, igfbp3*, and *igfbp6a*) increased in response to the Igf receptor inhibitor after 7 days of incubation (Figure 3B). This data shows that the late decrease of *igfbp1a, -3*, and *-6a* transcripts specifically depends on Fsh-triggered, Igf3-dependent signaling.

In fish, other mediators of Fsh effects are androgens, considering the strong steroidogenic potency of zebrafish Fsh, for example (6). While steroid-mediated effects were neutralized by including trilostane in the incubation medium in experiments with Fsh, the next set of experiments aimed at investigating potential androgen effects. 11-KT (200 nM) upregulated the expression of *igfbp2a* and *igfbp5b*, while *igfbp6a* was downregulated after 7 days but not after 3 days of incubation (Figure 3C). *Igfbp1a, 3*, and *6a* transcript levels did not response to 11-KT after 3 or 7 days of incubation (data not shown). Since *igf3* expression also responds to 11-KT (9) and since *igfbp* transcript levels responded to 11-KT after 7 days, the experiment was repeated in the presence of the Igf receptor inhibitor for 7 days. However, the effects of 11-KT on the transcript levels of *igfbp*s did not change in the additional presence of the Igf receptor inhibitor (data not shown), suggesting that the 11-KT effects were not mediated by Igf3.

### Igf3 Effects on Spermatogonial Development

**Figure 4:**
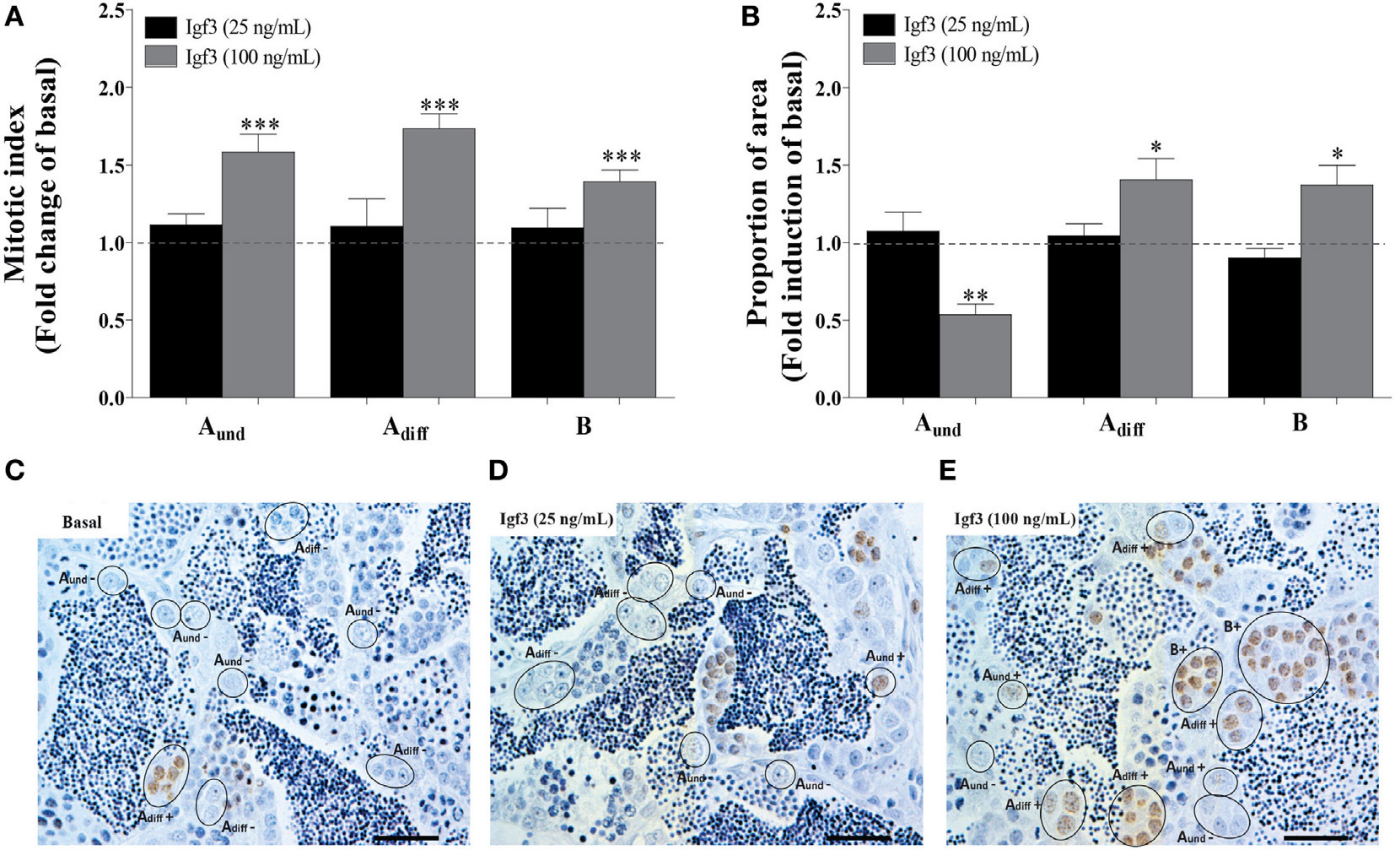
Effect of 25 or 100 ng/mL Igf3 on spermatogonial proliferation and proportion of area after 3 days of primary testis tissue culture. **(A)** Mitotic index of type A_und_, type A_diff_, and type B spermatogonia in the presence of 25 ng/mL (black bars) (*n* = 6) or 100 ng/mL Igf3 (gray bars) (*n* = 7). **(B)** Proportion of section surface area occupied by cysts containing type A_und_, type A_diff_, or type B spermatogonia, following exposure to 25 ng/mL (black bars) (*n* = 6) or 100 ng/mL Igf3 (gray bars) (*n* = 7). **(C–E)** Immunocytochemical detection of BrdU in sections of zebrafish testis incubated under basal conditions **(C)** or in the presence of 25 ng/mL **(D)** or 100 ng/mL Igf3 **(E)** for 3 days, showing BrdU positive (+) and negative (−) A_und_, A_diff_, and B spermatogonia. Bars, 25 µm. Dotted lines in A and B represent the mean values of the control groups (absence of Igf3). Asterisks indicate significant differences (*P < 0.05; **P < 0.01; ***P < 0.001) compared to the respective control group.

Exposure to 25 ng/mL Igf3 did not modulate the mitotic index and proportion of spermatogonia after 3 days of incubation (Figures 4A,C,D), different from a higher concentration of Igf3 (100 ng/mL) that increased the mitotic indices of all spermatogonia (Figures 4A,E). The proportion of area occupied by A_und_ was reduced, while the one for A_diff_ and B spermatogonia increased in the presence of 100 ng/mL of Igf3 for 3 days (Figure 4B).

### A Subthreshold Dose of Igf3 Becomes Active in the Presence of an Igfbp Inhibitor

**Figure 5:**
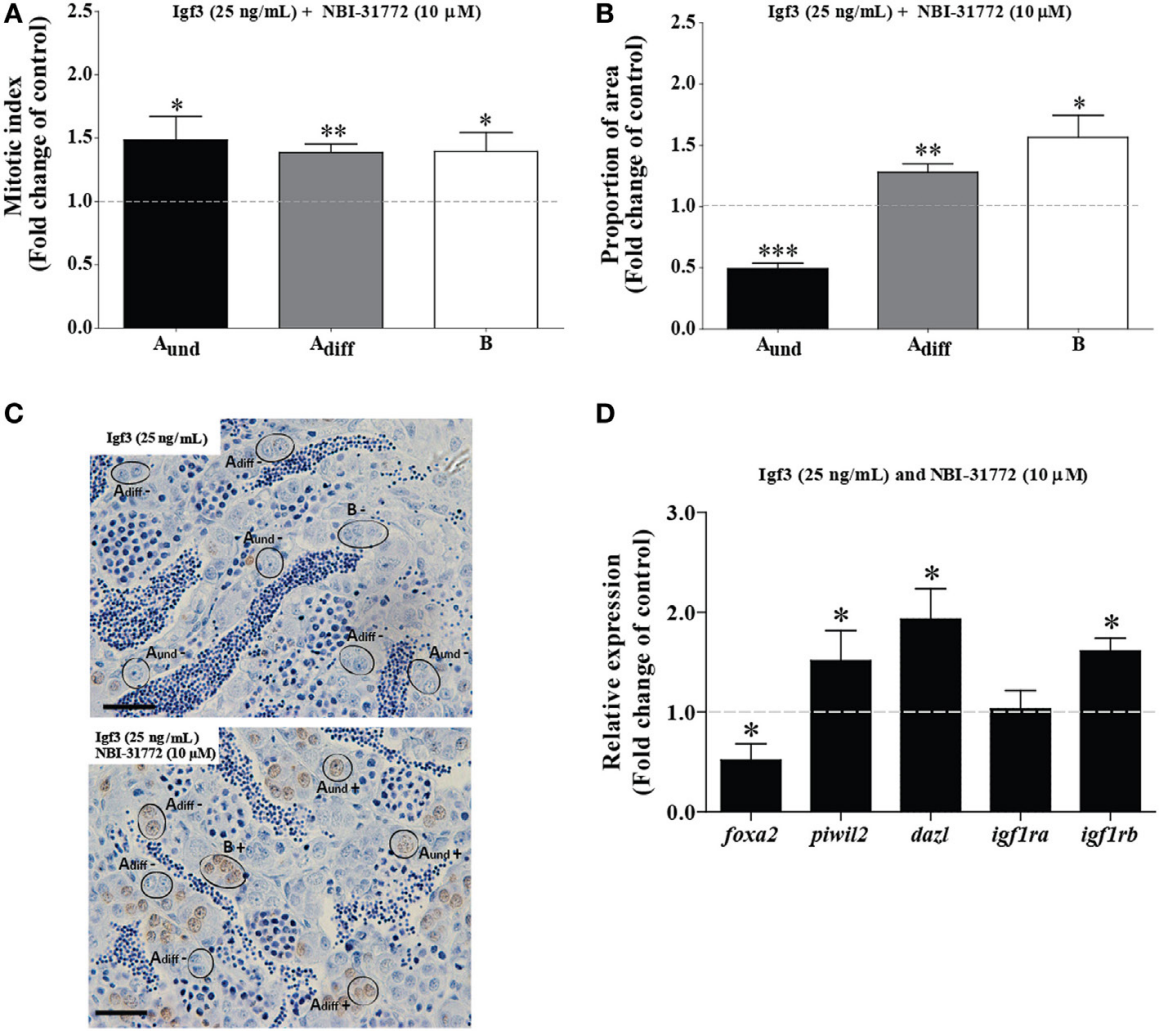
Effect of 25 ng/mL Igf3 in the presence of an IGF-binding protein inhibitor on spermatogonial proliferation and proportion of area after 3 days of primary testis tissue culture. **(A)** Mitotic indices of type A_und_, type A_diff_, and type B spermatogonia in the presence of Igf3 alone (25 ng/mL) (dotted line; control condition) or in combination with 10 µM NBI-31772 (bars) (*n* = 6). **(B)** Proportion of section surface area occupied by cysts containing type A_und_, type A_diff_, or type B spermatogonia in the presence of Igf3 alone (25 ng/mL) (dotted line; control condition) or in combination with 10 µM NBI-31772 (bars) (*n* = 6). **(C)** Immunocytochemical detection of BrdU in sections of zebrafish testis incubated with 25 ng/mL alone (upper panel; control condition) or in combination with 10 µM NBI-31772 (lower panel) for 3 days showing BrdU positive (+) and negative (−) A_und_, A_diff_, and B spermatogonia. Bars, 25 µm. **(D)** Gene expression analysis in adult zebrafish testis after 3 days of tissue culture in the presence of Igf3 (25 ng/mL) (represented by a dotted line) or in combination with 10 µM NBI-31772 (bars) (*n* = 7). Results are presented as fold changes with respect to the control group (25 ng/mL Igf3). Asterisks indicate significant differences (*P < 0.05; **P < 0.01; ***P < 0.001) between groups.

In order to better understand Igf3 signaling and its modulation by Igfbps in regulating spermatogenesis, we used NBI-31772, an inhibitor of Igf-Igfbp interaction. We asked if a low concentration of Igf3 not eliciting effects by itself (25 ng/mL; see Figure 4), does modulate BrdU incorporation and the proportion of section area occupied by spermatogonia when NBI-31772 was present as well. Indeed, the mitotic indices of all types of spermatogonia increased after 3 days of incubation in response to Igf3 and NBI-31772 (Figures 5A,C); also, the proportion of section surface area occupied by type A_diff_ and B spermatogonia increased, while the one for A_und_ decreased (Figure 5B). In parallel experiments, we quantified the transcript levels of selected genes to complement morphological with molecular data. Considering germ cell marker transcripts, *foxa2* (a potential marker for undifferentiated spermatogonia) transcript levels decreased, whereas *dazl* [expressed by B spermatogonia and primary spermatocytes (30)] and *piwil2* [expressed by all germ cells except A_und_ and spermatozoa (29)] expression was upregulated in the presence of Igf3 and NBI-31772 (Figure 5D), suggesting that the total number of germ cells has increased, associated with a shift from undifferentiated spermatogonia to B spermatogonia and spermatocytes. Transcript levels of *igf1rb* were also upregulated significantly (Figure 5D).

### Igfbps Upregulated by Hormones Support Spermatogonial Differentiation

**Figure 6:**
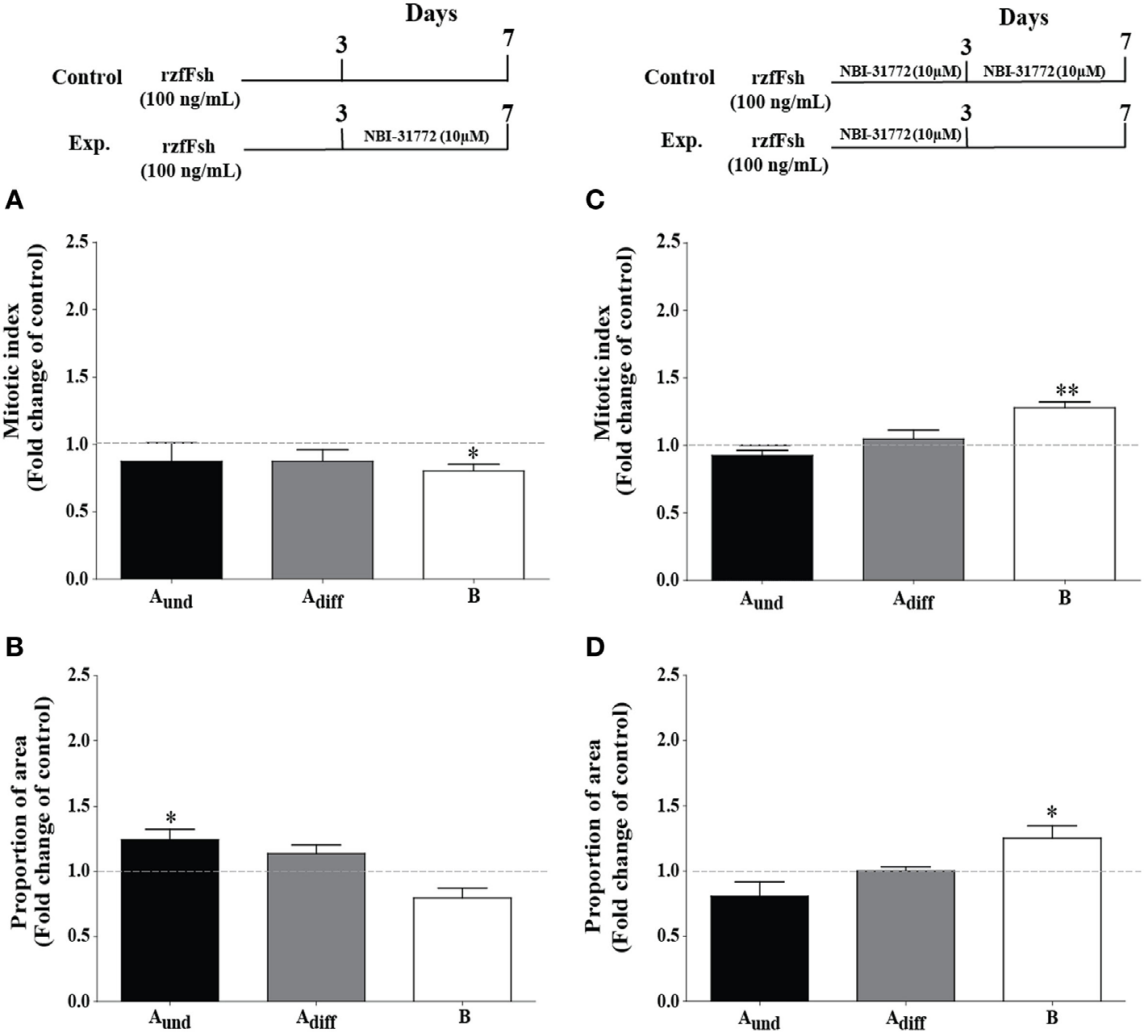
Effect of the presence (left panel) or absence (right panel) of an IGF binding protein inhibitor on follicle-stimulating hormone (Fsh)-stimulated spermatogonial proliferation and proportion of area after 7 days of primary testis tissue culture. **(A)** Mitotic indices and **(B)** proportion of area occupied by cysts containing type A_und_, type A_diff_, and type B spermatogonia in the presence of Fsh (100 ng/mL) for 7 days (control group, represented by stippled line) or 3 days in the presence of Fsh (100 ng/mL) and the remaining 4 more days in the additional presence of 10 µM NBI-31772 (experimental group); a schematic representation of the experimental setup is shown in the upper left panel (*n* = 6). **(C)** Mitotic indices and **(D)** proportion of area occupied by cysts containing type A_und_, type A_diff_, and type B spermatogonia in the presence of Fsh (100 ng/mL) and 10 µM NBI-31772 for 7 days (control group, represented by stippled line) or 3 days under the same conditions and the remaining 4 more days in the presence of Fsh (100 ng/mL) only (experimental group); a schematic representation of the experimental setup is shown on the upper right panel (*n* = 7). The production of biologically active steroids by Fsh was blocked by trilostane (25 µg/mL) in all cases. Results are presented as fold changes with respect to the control group. Asterisks indicate significant differences (*P < 0.05; **P < 0.01) between groups.

Previous work has shown that blocking Igf binding to Igfbps by NBI-31772 during 4 days of incubation with Fsh resulted in a strong pro-differentiation signal for spermatogonia and depleted undifferentiated spermatogonia (23), suggesting that Igfbps mainly restricted Igf3 bioactivity. However, the present time course and dose response experiments also showed that Fsh and 11-KT, two hormones promoting germ cell differentiation, can upregulate two *igfbp* transcripts with a delay of at least 3 days. It therefore seems possible that these Igfbps can support Igf3 bioactivity and contribute to the pro-differentiation signaling of Fsh and 11-KT. When the Igfbp inhibitor NBI-31772 was present only during the last 4 days of incubation (Figures 6A,B), when *igfbp2a* and *-5b* transcripts were upregulated by Fsh (Figures 2D,E) or 11-KT (Figure 3), the mitotic indices of A_und_ and A_diff_ did not change, whereas the one for type B decreased in response to Fsh (Figure 6A). The section surface area occupied by A_und_ increased, while the one for type B spermatogonia decreased in the presence of Fsh in combination with NBI-31772 during the last 4 days (Figure 6B). These observations suggest that blocking the “late rising” Igfbps partially inhibited spermatogonial differentiation. Inversing the experimental setting (i.e., NBI-31772 was only absent during the last 4 days of incubation) showed that the mitotic index and proportion of surface area of type B spermatogonia increased (Figures 6C,D). Under these conditions, the “early decreasing” Igfbps were blocked from the start of Fsh exposure, and the “late rising” Igfbps were allowed to bind Igf ligands. This resulted in a stronger pro-differentiation effect of Fsh, in particular for the type B spermatogonia (Figure 6D).

## Discussion

### Regulation of the igfbp Expression in Zebrafish Testis by Fsh and Downstream Mediators

More than 90% of the circulating IGF is bound to IGFBPs (32); hence, locally produced IGFBPs seem primarily involved in modulating locally produced IGF bioactivity. The recently discovered Igf3 is prominently [e.g., zebrafish (33)], in certain species preferentially (22), expressed in gonadal tissue of adult fish (34–37). Previous studies showed that one possibility for Fsh to stimulate the differentiating proliferation of type A spermatogonia in an androgen-independent manner is to release Igf3 (9). This Fsh effect was strengthened, leading to a partial depletion of type A_und_ spermatogonia, by blocking Igfbps during a 4-day culture period, suggesting that Igfbps protected A_und_ from excessive differentiation *via* Fsh-stimulated Igf3 release (23). These recent studies highlight the importance of the Igf signaling system in modulating zebrafish spermatogenesis. Here, we report that Fsh, next to regulating *igf3* and *igfbp1a* expression, modulated the expression of four other *igfbp*s. While *igfbp1a, igfbp3*, and *igfbp6a* transcript levels were downregulated quickly by Fsh, or more slowly by Igf3 or 11-KT, the expression of *igfbp2a* and *igfbp5b* increased with a delay of at least 3 days in response to Fsh or 11-KT. Information on the regulation of *igfbp* transcript levels is scarce, and few studies have addressed *igfbp* expression in gonads. In rat, *Igfbp2, 3*, and *4* transcripts have been detected in LCs and seminiferous tubules (38) and FSH reduced *Igfbp3* transcript levels in hypophysectomized rats (39). *Igfbp2-6* were found in sheep testis in association with high *Igf1* levels (40). In rainbow trout testis, the expression of *igfbp6* was upregulated by Fsh and its levels slightly decreased in the additional presence of trilostane, suggesting that both Fsh and androgens increased *igfbp6* expression in this species (41). To our knowledge, our study is the first to investigate dose and time effects of Fsh, revealing a dynamic modulation of *igfbp* transcript levels that is apparently relevant for modulating Igf3 bioactivity in zebrafish testis.

Igf and steroid hormones modulated *igfbp* expression in non-gonadal tissues in fish (42, 43). We examined if Igf3 or 11-KT, both mediators of Fsh bioactivity, were involved in the regulation of the Fsh-modulated testicular *igfbp*s. The transcript levels of *igfbp1a, -3*, and *-6a* were modulated in the presence of Igf3 or in response to Fsh and an Igf1r inhibitor after 7 days of incubation, suggesting that Igf3 is a downstream mediator of Fsh on *igfbp* expression and that the faster response induced by Fsh used a different mechanism than the delayed response mediated by Igf3.

**Figure 7:**
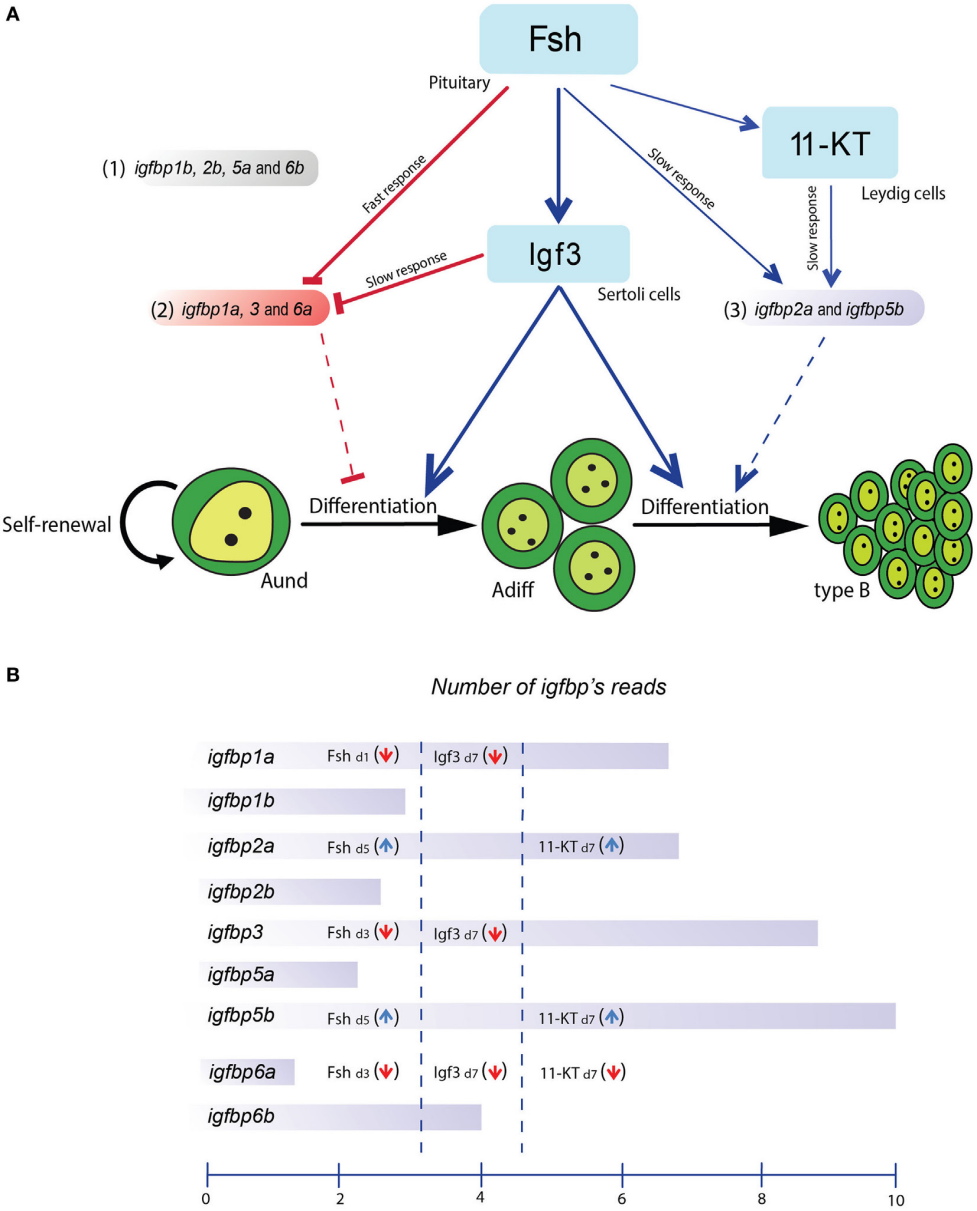
Schematic representation of the effects of follicle-stimulating hormone (Fsh) and two downstream mediators, Igf3 and 11-KT, on IGF-binding protein (*igfbp*) transcript levels and potential roles of Igfbps in adult zebrafish testis. **(A)** Fsh rapidly reduced transcript levels of subgroup 2 (consisting of *igfbp1a, -3*, and *-6a*) and increased the expression/release of Igf3 and 11-ketotestosterone (11-KT). Igf3 also reduced the transcript levels of subgroup 2 members whereas those of subgroup 3 (consisting of *igfbp1b, -2b, -5a*, and *-6b*) were increased by Fsh and 11-KT. The transcript levels of subgroup 1 (consisting of *igfbp2a* and *-5b*) were not modulated in the present experiments. **(B)** Schematic representation of the *igfbp* transcript levels and their hormonal regulation in zebrafish testis tissue. The representation of the *igfbp* transcript amounts reflects read numbers from RNAseq data (Crespo, Bogerd, and Schulz, unpublished data) from 5 testes of normal adult males. The mean read numbers were transformed using the logarithm to the base 2, i.e., the scale covers a 1024-fold (2^10^) difference in average read numbers.

However, since Fsh increases Igf3 release (9), we can expect a fast drop of *igfbp* transcript levels induced by Fsh, and a continued suppression of transcript levels mediated by Igf3. The androgen 11-KT, on the other hand, selectively increased *igfbp2a* and *-5b* but reduced *igfbp6a* transcript levels after 7 days of incubation. Based on these results, the *igfbp*s produced in the testis can be grouped in three categories: (1) non-responding to Fsh, Igf3, or 11-KT, (2) downregulated by Fsh, Igf3 or 11-KT, and (3) upregulated by Fsh and 11-KT but not by Igf3 (Figure 7).

The present data not only show that *igfbp* transcript levels respond to Fsh and downstream mediators but also open the possibility that Igfbps exert differential effects on testicular Igfs, potentially restricting or supporting Igf signaling. Still, the *igfbp*s not modulated by Fsh, Igf3 or 11-KT should not be disregarded. In addition, Igfbps can act in an Igf-independent manner in mammals (44). Also in zebrafish, Igfbp3 blocked bone morphogenetic protein (Bmp) signaling by binding Bmp2a during embryonic development (45). Zebrafish Igfbp3, -5a, and -5b were localized also in the nucleus of U2O2 and HEK 293 cells (45, 46). Igfbp5a and -5b differ considering that Igfbp5b, but not -5a, shows transactivation activity in zebrafish (46). Since *igfbp5b* transcript levels are ~250-fold higher than those of *igfbp5a*, the latter also not being regulated by Fsh or 11-KT, it seems possible that Igfbp5b might be the more relevant form for potential nuclear functions in the testis. However, in general, the functional significances of nuclear Igfbps are still not well understood.

### Fsh-Modulated igfbps Can Support or Inhibit Spermatogonial Differentiation in Zebrafish Testis

Previous studies have suggested that Igfbp can inhibit or enhance Igf action. While IGFBP1 and -6 generally inhibited IGF actions, IGFBP2-5 can inhibit or potentiate the IGF action, depending on the cell or tissue type, or on the physiological or experimental context (24). Due to the important role of Igf in muscle, many studies on extrahepatic IGFBP function addressed this tissue. In vascular smooth muscle cells, IGFBP2 and IGFBP4 exert an inhibitory effect on IGF1-induced DNA synthesis, while IGFBP5 potentiates the mitogenic effect of IGF1 (47, 48). Knock down of IGFBP5 impairs myogenesis and downregulated *IGF2* expression in cultured myoblast cells (49). Administration of IGFBP5 in combination with a low concentration of IGF2 restored *IGF2* expression and myogenic differentiation, whereas a non-functional IGFBP-5 or an IGF analog that activates the IGF1R but cannot bind IGFBPs, had no or a limited effect, respectively (49). These differential Igfbp effects seem to be conserved in fish. The transition from zero growth (achieved by food restriction) to fast growth involves upregulation of *igf1, igfbp4*, and -*5b* in Atlantic salmon skeletal muscle (50). Moreover, primary cultures of Atlantic salmon myogenic satellite cells (stem cells in muscle) respond to amino acids and/or Igf1 by expressing high levels of *igf1* and *igf2*, and *igfbp4, -5a*, and *-5b* (42). Similarly, upregulation of *igfbp2, igfbp4, igfbp5*, and *igf1* was recorded during muscle growth recovery after the end of a starvation period in rainbow trout (51). A dual role for the Igfbps has also been suggested in the regulation of zebrafish muscle growth and differentiation (12). Here, we have started exploring the potentially dual role of Fsh-regulated *igfbps* on zebrafish spermatogenesis.

Stimulatory effects of Igf3 on spermatogonial proliferation have been reported previously (9). The latter study did neither examine potential effects on type B spermatogonia nor on the volume fractions occupied by the different spermatogonia. We report here that Igf3 (100 ng/mL) promoted the proliferation of all spermatogonial cell types and also increased the areas occupied by A_diff_ and B spermatogonia while reducing the one occupied by A_und_ spermatogonia. This suggests that Fsh-stimulated Igf3 release promotes differentiation of A_und_ into A_diff_ and further into B spermatogonia. Our study also reports several findings as regards Igfbp functions in testis physiology, based on examining the effects of NBI-31772 on Igf3 activity. For example, blocking Igfbps increased the biological activity of a sub-threshold dose of Igf3, and Igf3 release stimulated by either Fsh or thyroid hormone preferentially promoted differentiation of A_und_ spermatogonia when Igfbps were blocked (23). These findings suggest that Igfbps protect the pool of A_und_ spermatogonia against excessive differentiation driven by high levels of Igf3. Our data moreover indicate that this protective effect may be mediated by the three *igfbp*s rapidly down regulated by Fsh. Interestingly, the response to blocking Igfbps included upregulation of the expression of *igf1rb*. As mentioned above, zebrafish testis tissue expresses both *igf1 receptor* genes and the expression of *igf1rb* was previously upregulated under experimental conditions promoting spermatogonial proliferation (30). Altogether these results suggest that Igf3-mediated stimulation of spermatogonial proliferation and differentiation that is enhanced by blocking inhibitory Igfbps may involve upregulation of *igf1 receptor* expression.

In addition to the three *igfbp* transcripts being downregulated by Fsh, Igf3, or 11-KT, two other family members (*igfbp2a* and *-5b*) were upregulated in a delayed manner by Fsh or 11-KT. Blocking and de-blocking experiments suggested that the “late-rising” binding proteins facilitate pro-differentiation effects of Igf. Therefore, we propose that the concept of specific Igfbps either limiting or supporting Igf bioactivity, is also valid for testis tissue, where Fsh, but also downstream mediators (Igf3 and androgens), modulate *igfbp* gene expression. While Fsh and Igf3 or Fsh and 11-KT have similar effects as regards the direction of change, they exert their effects on *igfbp* transcript levels with differences in the time course, suggesting the use of different mechanisms to modulate *igfbp* gene expression. This may allow more sustained effects. Both, the acute as well as the delayed effects of Fsh *via* the Igf signaling system affected all spermatogonial cell types (A_und_, A_diff_, and B), suggesting that Fsh promotes spermatogonial development in a broad sense, making use of the Igf signaling system to generate different signals over time to different germ cell generations.

Several other signaling systems are also modulated by Fsh, next to the Igf/Igfbp system (11). It will be interesting to address in future studies the differentiation of the response to Fsh in space, e.g., by examining if spermatogenic cysts containing germ cells in different stages of development respond differently to a given Fsh challenge with respect to the expression of different *igfbp* transcripts or transcript amounts. Also in context with our previous observations (23), we propose that Igfbps negatively modulate the activity of Igf3 in the presence of a comparatively weak stimulator of Igf3 release, T_3_, whereas when Fsh is present, Igfbps restricting Igf3 action (Igfbp1a, Igfbp3, and Igfbp6a) are rapidly suppressed, while the availability of Igfbps supporting Igf3 action (Igfbp2a and Igfbp5b) increases after a lag phase of 3–5 days, when suppression of the inhibitory Igfbps is also supported by downstream effectors of Fsh, such as Igf3 and androgens (Figure 7).

In conclusion, we have shown that of the nine *igfbp*s expressed in zebrafish testis tissue, five are selectively modulated by Fsh and two Fsh downstream mediators (Igf3 and 11-KT) to promote spermatogonial differentiation. We also report that the pro-differentiation effect of Igf3 is reinforced by blocking the binding of Igf to the rapidly downregulated Igfbps, supporting the role for certain Igfbp as protecting A_und_ from excessive differentiation in response to Igf3.

## Ethics Statement

All experiments carried out in this study followed the Dutch National regulations for animal care and use in experimentation, and the experimental protocols have been submitted to, and were approved by, the Utrecht University Experimental Animal Committee (2015.I.857.013 and AVD108002015333).

## Author Contributions

DS, HK, and DC conducted all the experiments and analyzed the data. DS, JB, and RS designed the experiments and wrote the manuscript.

## Conflict of Interest Statement

The authors declare that the research was conducted in the absence of any commercial or financial relationships that could be construed as a potential conflict of interest.
