# Supplementary material for: Follicle-Stimulating Hormone Regulates igfbp Gene Expression Directly or via Downstream Effectors to Modulate Igf3 Effects on Zebrafish Spermatogenesis
Source: Front Endocrinol (Lausanne). 2017 Nov 20;8:328. doi: 10.3389/fendo.2017.00328 (PMC5702253; doi:10.3389/fendo.2017.00328)
Supplement: Supplementary file 1 [file data_sheet_1.docx]

***Supplementary Material***

**Fsh directly and indirectly modulates Igf3 effects on spermatogenesis by regulating *igfbp* expression in adult zebrafish testis**

Diego Safian, Henk van der Kant, Diego Crespo, Jan Bogerd and Rüdiger W. Schulz^*^

*** Correspondence:**

Corresponding Author

Rüdiger W. Schulz

Reproductive Biology Group, Division of Developmental Biology

Institute of Biodynamics and Biocomplexity, Department of Biology

Faculty of Science

Utrecht University

Kruyt Building (Room W-606), Padualaan 8, 3584 CH Utrecht, The Netherlands.

***Fig. 1***

***
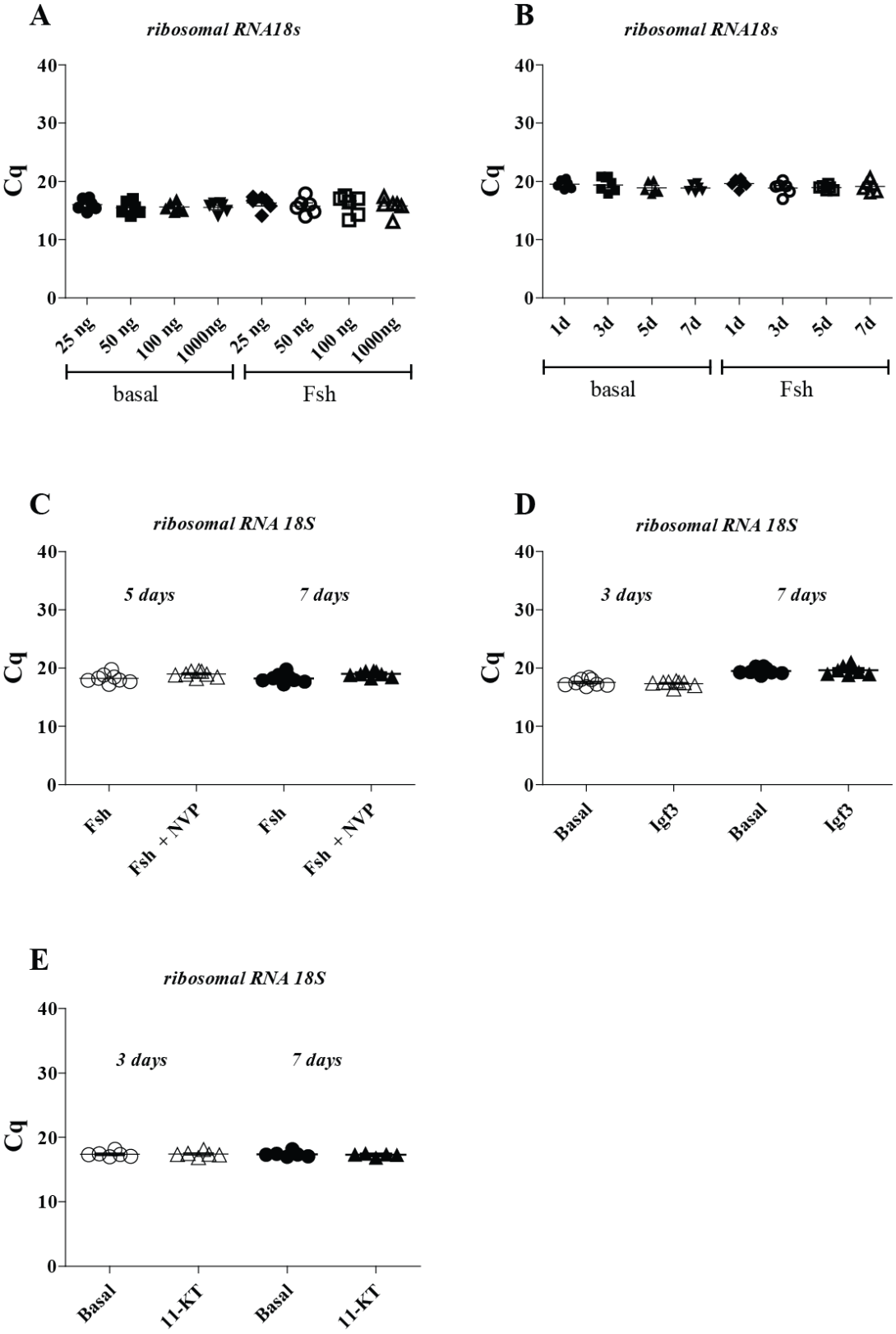
***

**Figure 1.** Quantification cycles (Cq) of *ribosomal RNA* *18S* under basal conditions and in the presence of 25, 50, 100 or 1000 ng recombinant zebrafish Fsh per mL (n=6 for all concentrations) (A), basal conditions or 100 ng/mL Fsh for 1, 3 ,5 and 7 days ((n = 5-7) (B), in the presence of 100 ng/mL Fsh with or without Igf1r receptor inhibitor NVP-AEW541 (10 µM; NVP) for 5 or 7 days (n = 8) (C), basal conditions or in the presence of zebrafish Igf3 (100 ng/mL) for 3 or 7 days (n = 8) (D) or basal conditions or in the presence of 11-KT (200nM) for 3 (n = 7) or 7 (n = 6) days (E).

***Fig. 2***

***
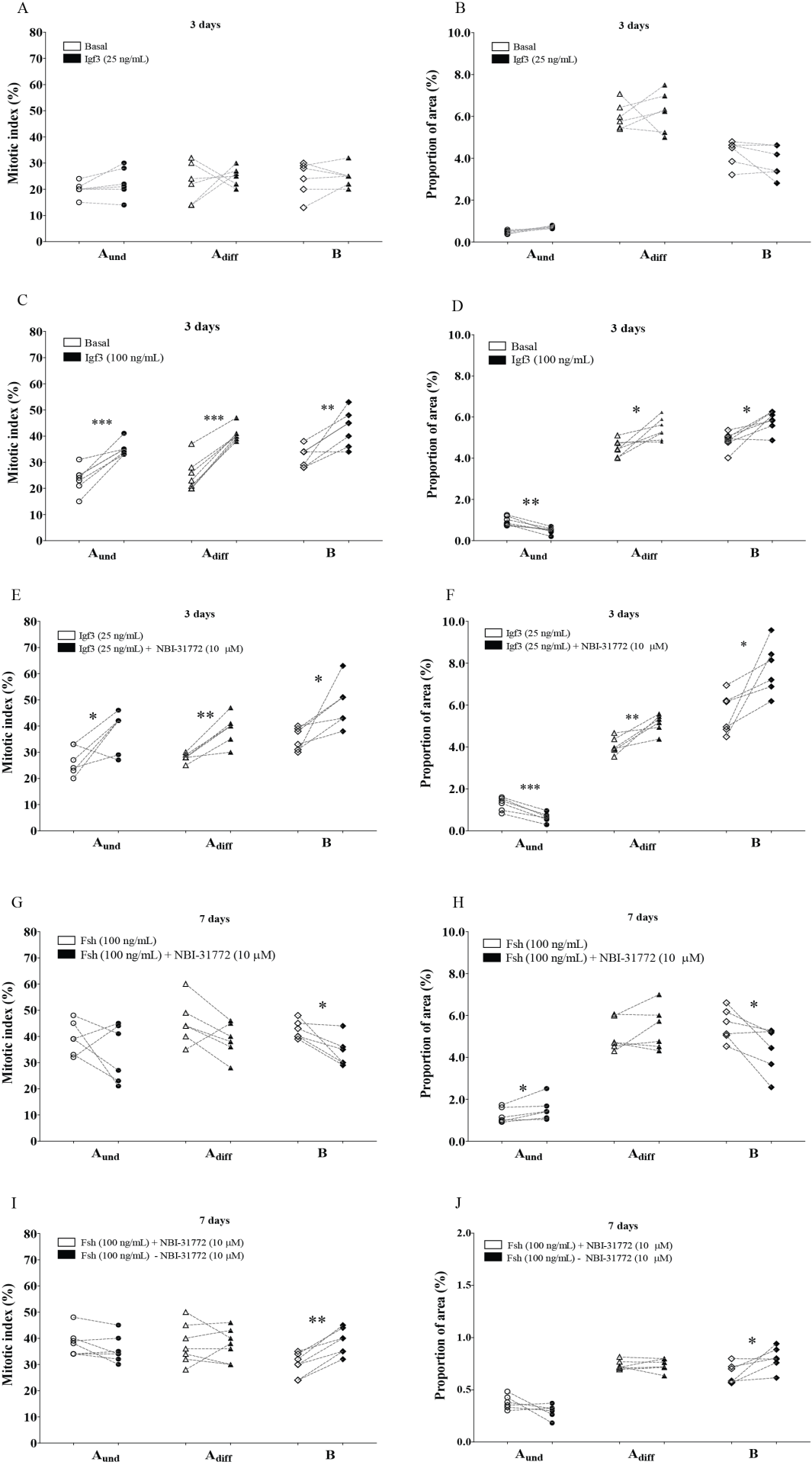
***

**Figure 2.** Mitotic index and proportion of area in testis incubated in basal conditions or in the presence of Igf3, Igf3 and NBI-31772 or Fsh and NBI-31772. (A) Mitotic indices and (B) proportion of area of type A_und_, type A_diff_ and type B spermatogonia in basal conditions or in the presence of Igf3 (25 ng/mL) (n=6) for 3 days. (C) Mitotic indices and (D) proportion of area of type A_und_, type A_diff_ and type B spermatogonia in basal conditions or in the presence of Igf3 (100 ng/mL) (n=7) for 3 days. (E) Mitotic indices and (F) proportion of area of type A_und_, type A_diff_ and type B in presence of Igf3 (25 ng/mL) or Igf3 (25 ng/mL) in combination with 10 µM NBI-31772 (n=6). (G) Mitotic indices and (H) proportion of area of type A_und_, type A_diff_ and type B spermatogonia in presence of Fsh (100 ng/mL) for 7 days or Fsh (100 ng/mL) in combination with 10 µM NBI-31772 in the last 4 days of a 7 days incubation (n=7). (I) Mitotic indices and (J) proportion of area of type A_und_, type A_diff_ and type B spermatogonia in presence of Fsh (100 ng/mL) and 10 µM NBI-31772 for 7 days or only Fsh (100 ng/mL) in the last 4 days of a 7 days incubation (n=7). The production of biologically active steroids by Fsh was blocked by including trilostane (25 µg/mL). Dotted lines connect testes from the same animal. Asterisks indicate significant differences (* *P*<0.05; ** *P*<0.01; *** *P*<0.001) between groups.
